# Buffalo Medical College

**Published:** 1850-10

**Authors:** 


*Buffalo Medical College.*—The preliminary term of instruction in this Institution commences on the 2d inst., and continues to the first Wednesday in November, the date of the commencement of the regular term. This gives a preliminary term of *five* weeks' duration. The regular term continues *sixteen* weeks.

